# Long-term outcome of the IMZ implant system: a retrospective clinical study with a follow-up between 23 and 34 years

**DOI:** 10.1186/s40729-022-00452-0

**Published:** 2022-12-01

**Authors:** Christoph Mautsch, Stefan Wolfart, Walter Mautsch, Anne Barbara Rittich

**Affiliations:** Department of Prosthodontics and Dental Materials, Medical Faculty, RWTH Aachen University, Pauwelsstr. 30, 52074 Aachen, Germany

**Keywords:** Marginal bone loss, Dental implants, Long-term follow-up, IMZ implant system, Risk factors, Peri-implantitis

## Abstract

**Purpose:**

To evaluate the radiographic and peri-implant outcomes of intramobile cylinder implants (IMZs) and the feasibility of long-term follow-up studies after nearly 30 years.

**Methods:**

Of the 94 patients treated with IMZ implants between 1981 and 1995, 39 patients were successfully contacted (contact group, CG), of which 15 patients with a total of 32 implants agreed to participate in the present follow-up study (clinical evaluation group, CEG). The overall implant survival rate was calculated. Information on implant status and oral and general health data was collected. Marginal bone level was evaluated and then compared to the patients’ baseline data. Possible risk factors for peri-implantitis were also identified.

**Results:**

In total, 16 implants in seven patients were lost, amounting to an overall survival rate of 79.5% after 30 years with a mean follow-up time of 24 ± 10 years (CG). Eight patients were treated with bar-retained mandibular overdentures and seven patients had fixed partial dentures. After a mean observation time of 29 ± 3 years, the surviving implants showed a peri-implantitis rate of 9.4% with a mean marginal bone loss of 2.5 ± 1.8 mm (CEG). No significant correlation between peri-implantitis and possible risk factors could be found.

**Conclusions:**

Long-term follow-up studies with acceptable response rates after nearly 30 years are not feasible. Contact was only possible with 41% of the patients. This contact group showed a high implant survival rate. Due to the retrospective study design, additional risk factors could not be considered in a conclusive analysis.

**Graphical Abstract:**

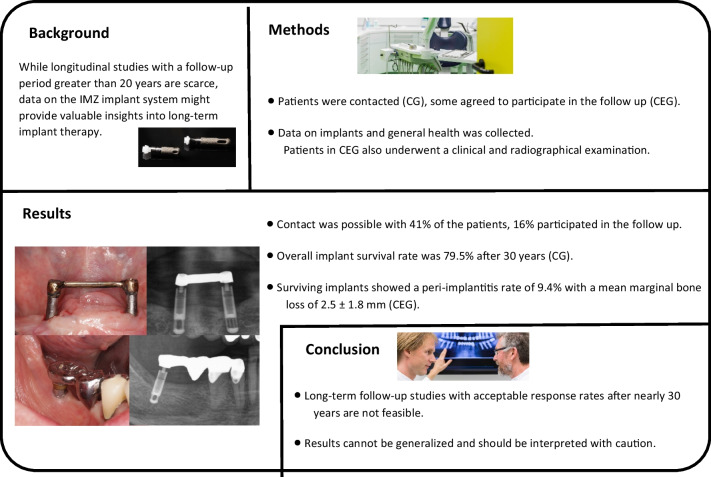

## Background

The discovery of osseointegration by Branemark in 1969 [1] opened up a multitude of new possibilities for restoring health, esthetics, and function in edentulous patients and those with extensive damage to their dentition. Therefore, implant therapy has revolutionized dental practice. Along with the implant ad modum Branemark, intramobile cylinder implants (IMZs) were among the first fixtures used in implant therapy. The IMZ implant system was particularly popular in the 1980s and the early 1990s, before it was replaced by the Camlog implant system in the late 1990s. The key component of the IMZ implant system is the intramobile element (IME), whose purpose is to simulate the viscoelasticity of the periodontal ligament and reduce the forces transmitted to the marginal bone–implant interface [2]. Since the implant and IME are rigidly connected, the IME serves to reduce the displacement differential between the osseointegrated implant and a natural tooth while also impeding the intrusion of natural teeth, which can occur if a nonrigid interlock is used [3]. Several previous studies have reported excellent results on survival rate and radiographic and clinical data [3–7]. However, to date, longitudinal studies with a follow-up period greater than 10 years are scarce. Previous prospective studies with a follow-up period of up to 10 years have reported findings on the IMZ Implant system [6, 8, 9]. There are also a few additional retrospective studies reporting on IMZ implants after a period of more than 10 years, but as implants of significantly lower age were also included in those studies, the overall mean observation time was considerably shorter [7, 10]. A literature search in MEDLINE on the IMZ Implant system regarding follow-ups with a mean observational period of at least 5 years reveals a total of four studies of heterogenous design shown in Table 1. Further, additional three studies report results after up to 13 years of follow-up without specifying the mean observation time. Although all authors report on implant survival or success rates, data on peri-implant conditions such as marginal bone loss is hardly mentioned [2–11]. While existing data indicate high survival rates of over 90% and minimal marginal bone-level changes of less than 2 mm after a period of 10 years [6], the long-term outcome of the IMZ implant system is still unknown, as there are no studies reporting data after at least 20 years.

**Table 1:** Publications providing medium- and long-term data on the IMZ Implant System

Authors	Design	Mean observation time (years)	Patient selection	Type of restoration	Number of patients/implants	Drop-out (%patients/%implants)	Mean marginal bone loss (mm)	Peri-implantitis rate	Survival rate (implants)
Kirsch & Ackermann 1989	Retrospective	Not specified (1–10 years)	Patients with sufficient horizontal and vertical residual bone	SC, FDP, RDP	1401/3088	?/334 (?/10.8%)	Not specified	Not specified	97.8% (excluding drop out)
Spiekermann et al. 1995	Prospective	5.7	No special features	RDP	125/264	Not specified	2–6 (depending on IMZ-implant type)	Not specified	> 90% (cumulative after 5 years)
Noack et al. 1999	Retrospective	Not specified (1–13 years)	Treatment between 1984 and 1997	SC, FDP, RDP	527/1250	Not specified	Not specified	Not specified	81% (cumulative, after 10 years)
Willer et al. 2003	Prospective	Not specified (1–13 years)	No special features	SC, FDP, RDP	541/1250	89/136 (16.5%/10.9%)	Not specified	Not specified	82.4% (cumulative, after 10 years)
Meijer et al. 2003	Prospective	10	- Edentulousness for at least one year - Problems with retention and stability of the lower denture	RDP	29/58	1/2 (3.4%)	No mean values reported	Not specified	93% (percentage implants lost)
Meijer et al. 2009	Prospective	10	- Severely reduced mandible -Edentulousness - Problems with retention and stability of the lower denture	RDP	30/60	1/2 (3.3%)	1.4 ± 1.1	Not specified	93% (percentage implants lost)
Visser et al. (2016)	Prospective	15	- Treated between 1996 and 1999 -Edentulousness for at least 2 years - Functional problems	RDP	40/160	20/80 (50%)	Not specified	Not specified	88.7%-98.7% (percentage implants lost, range between subgroups)
Our Findings	Retrospective	28.3	- Treated between 1981–1995 - Availability of baseline radiographs	SC, FDP, RDP	94/199	79/167 (84%/83.9%)	2.5 ± 1.8	9.4%	79.5%

Notably, middle-aged or younger patients with multiple agenesis are often treated with endosseous implants and are reliant on the function of their implants for many decades. Considering these patients’ age, longer observation periods than those typically seen in 5- to 10-year follow-up periods are appropriate and needed. While many aspects of implant design have changed over the years, the overall principle and structure of endosseous implants have remained unaltered. Importantly, data on older implant systems might provide valuable insights into long-term implant therapy, even with the current systems. Therefore, the aims of the present retrospective study were: 1) to examine the feasibility of implant therapy follow-up examinations after a mean observation time of at least 20 years; 2) to investigate the outcome of IMZ implant therapy providing clinical and radiographical long-term results.

## Methods

### Patient selection

**Fig. 1:**
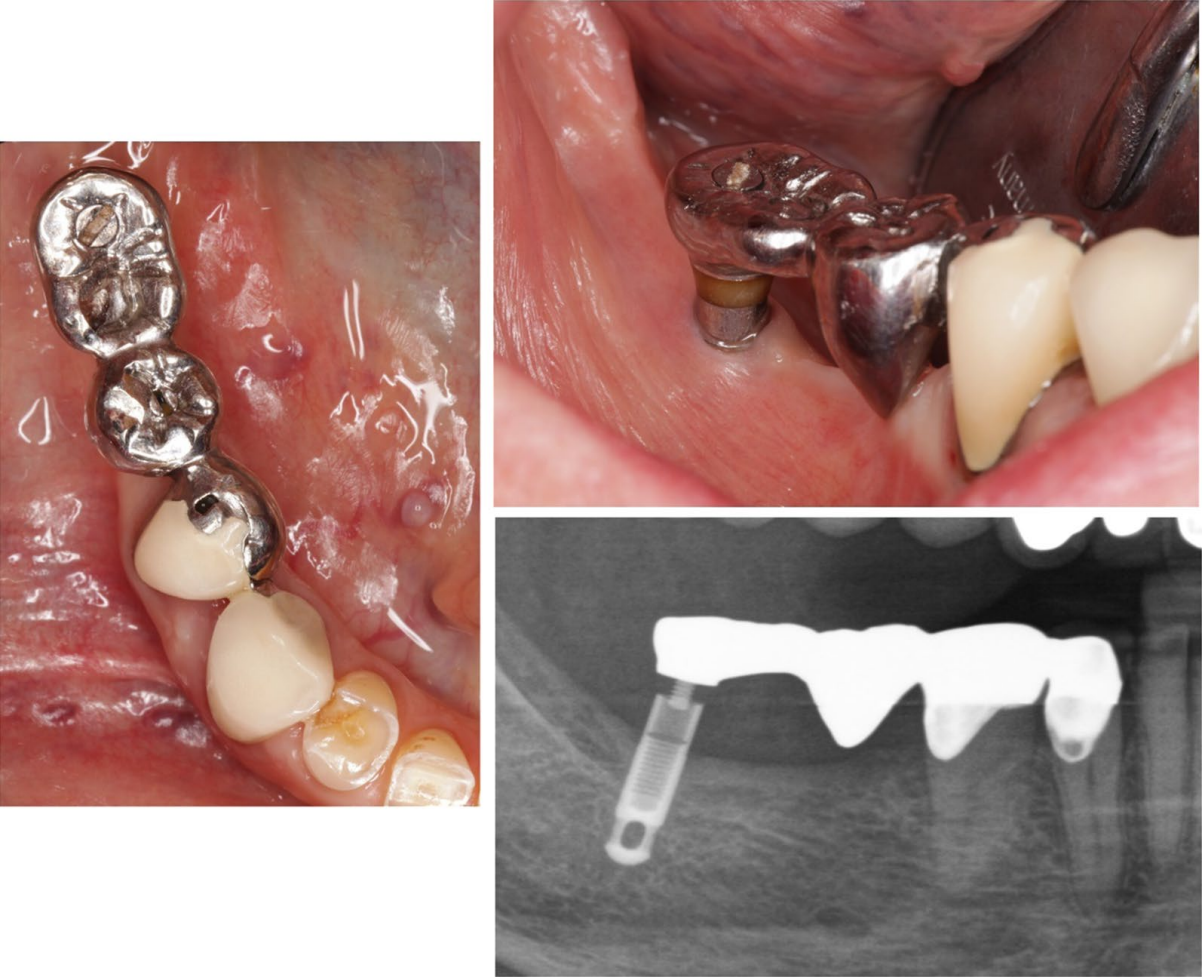
Clinical photographs and radiograph of a combined tooth–implant retained fixed partial denture in clinical examination group (CEG) after 26 years in situ. Note the radiolucent intramobile element (IME) between the IMZ implant and the superstructure as well as the transmucosal implant extension (TIE), which can be distinguished from the implant itself by a subtle radiolucent line.

**Fig. 2:**
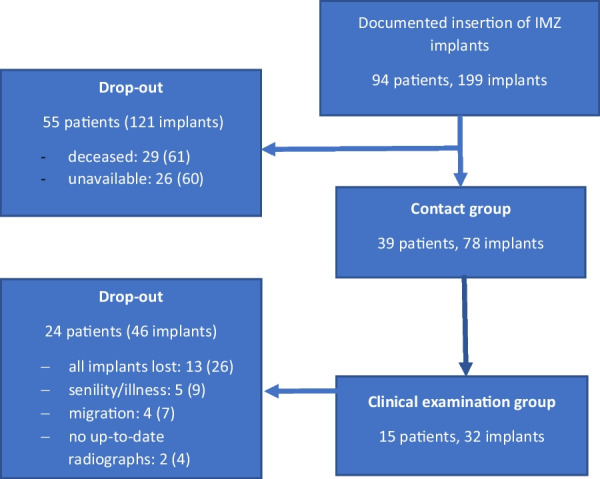
Flow diagram of patient identification

In this study, patients treated with intramobile cylinder implants (IMZ) at the Department of Prosthodontics of the University Hospital of RWTH Aachen between 1981 and 1995 were preselected. The only additional selection criteria were the availability of baseline radiographs**.** Of the 94 possible candidates, 29 patients died and 26 were unavailable. In total, 39 patients with 78 implants were successfully contacted. Information on general health and implant status was gathered through telephone interviews and mail questionnaires (contact group = CG). In addition, archived patient records were consulted to calculate the implant survival rate in the CG. After excluding four patients who had moved to a new address far from the study location, five patients who did not want to participate due to senility, and 13 patients who lost all of the IMZ implants originally inserted, 17 patients agreed to participate in a clinical examination. There was no indication for radiographic examination in two patients, thus a total of 15 patients with 32 implants were included in the present follow-up study (clinical examination group = CEG) (Figs. 1, 2).

### Clinical analysis

During the follow-up examination, which was conducted from May–July 2017, information on oral and general health was collected. Patients were asked if they had been previously diagnosed with periodontitis, diabetes, or osteoporosis. Additionally, data on smoking habits were collected. The pocket probing depth (PPD) was measured at four sites for each implant (mesial, labial, distal, and oral) using a calibrated periodontal probe. Additionally, the average probing depth for each individual implant was calculated. At the same time, the BOP Index (bleeding on probing) was evaluated for each of the four implant sites. The bleeding points for each implant were counted, amounting to a maximal Bleeding Index of 4 for each implant. The width of the attached mucosa was measured using the same periodontal probe. The presence of plaque was assessed using the index according to Mombelli et al. [12]. PIaque Index (PI) was also evaluated at four sites, but only the highest index value for each implant was used for the analysis. Furthermore, implants were categorized into two groups according to the type of restoration, to differentiate between implants in fixed and removable prostheses. Rates for implants diagnosed with peri-implantitis were calculated for the whole study population as well as for implants in the fixed and removable prostheses, using the case definition for peri-implantitis proposed in the Consensus report of the 2017 World Workshop on the Classification of Periodontal and Peri-Implant Diseases and Conditions. Implants with a marginal bone loss of ≥ 3 mm compared to baseline in combination with bleeding or suppuration on probing as well as probing depths of ≥ 6 mm were classified as peri-implantitis cases [13].

### Radiographic analysis

**Fig. 3:**
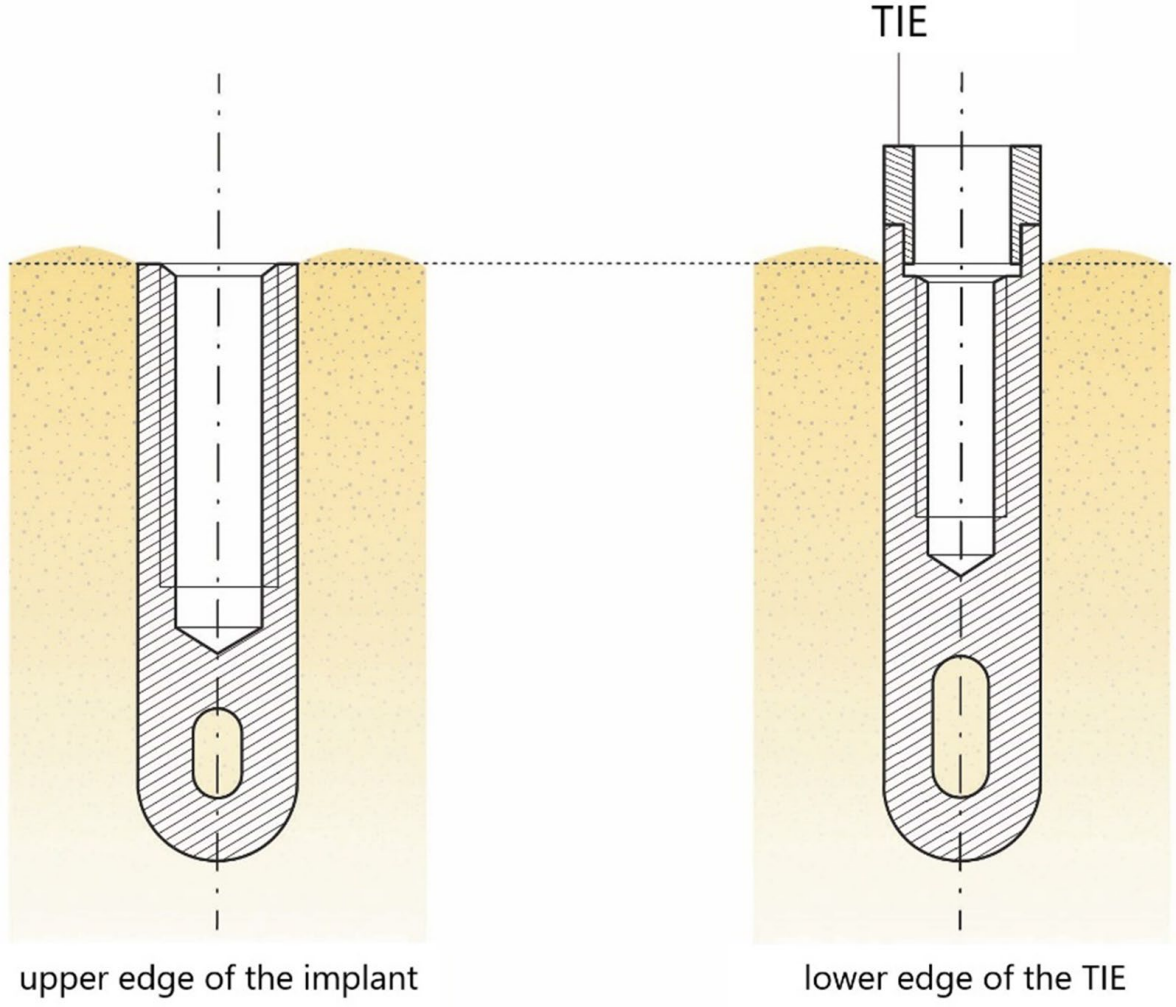
Reference points for radiographic measurements of the marginal bone level for IMZ implants with and without transmucosal implant extension (TIE). Implants were placed into the bone as far as these reference points

For the radiographic assessment, we compared the patient’s latest radiographs to their baseline data. Unfortunately, due to the long observation period, some archived patient health records were incomplete. As it was not possible to recollect all accurate baseline radiographs, we decided to include radiographs, panoramic radiographs, and standardized intra-oral radiographs taken up to 18 months after implant insertion as baseline. For the current analysis, radiographs collected within the last year of the follow-up examination were accepted. If there were signs of inflammation or peri-implantitis, radiographs were also obtained during the follow-up examination. Linear measurements were performed by two independent examiners with correction of the radiograph magnification using an implant of known length as the measuring gauge. The reference points were defined as shown in Fig. 3. Estimations for mean marginal bone (MBL) loss comparing bone levels at baseline and follow-up were performed mesially and distally as well as for each implant individually, same as with PPD.

### Statistical analysis

Descriptive statistics of both study groups were obtained by analyzing the frequency distributions of patient and implant characteristics. Possible implant site-dependent differences in marginal bone loss were analyzed using the Mann–Whitney *U*-test. Inter-rater reliability was assessed using the intraclass correlation coefficient. Analysis of possible patient risk factors with regard to the presence of peri-implant disease was performed using the Chi-squared test. Differences in clinical and radiographic findings between implants in fixed and removable prostheses were analyzed using the *t*-test and Mann–Whitney *U* test, whereas possible correlations between radiographic and clinical parameters were identified using the Spearman-Rho test. Values of all parameters were checked on a standard distribution using the Shapiro–Wilk test. Statistical analysis was performed using SPSS (Statistical Package Social Sciences, version 25, SPSS Incorporated, Chicago, IL, USA). In all tests, a significance level of 0.05 was chosen.

## Results

### Study data

The contact group with 39 patients and 78 implants consisted of 28 female and 11 male subjects with an average age of 74.7 ± 8.5 years. In total, six patients were diagnosed with diabetes, eight with osteoporosis, and 15 with periodontitis in the past, while 10 patients described themselves as smokers. Of the 78 implants, 56 were placed in the mandible. Most patients received implant treatment with 12 and 22 patients receiving one or two implants, respectively. In five cases, three or more implants were inserted.

In the examination group, a total of 15 patients with 32 implants attended follow-up. The study population consisted of 10 female and five male patients with an average age of 75.3 ± 1.5 years. Of the 32 included implants, 27 were placed in the mandible and five were inserted in the maxilla. Eight patients received implant treatment with two implants, while four patients received treatment with one or three implants.

### Implant survival rates

**Fig. 4:**
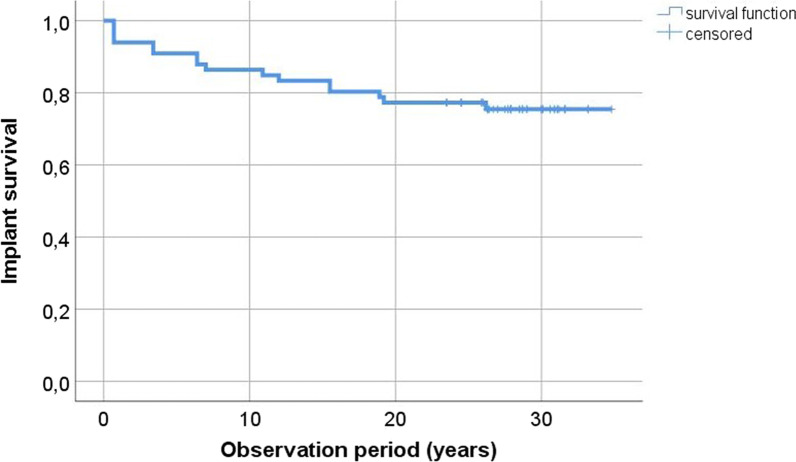
Kaplan–Meier curve representing implant survival in contact group (CG)

After a mean follow-up time of 24 ± 10 years a total of 16 implants in 7 patients were lost. The implant survival rate amounts to 79.5% in contact group (Fig. 4).

In the examination group, no implant was lost after an observation period of 29 ± 3 years.

### Clinical examination

**Fig. 5:**
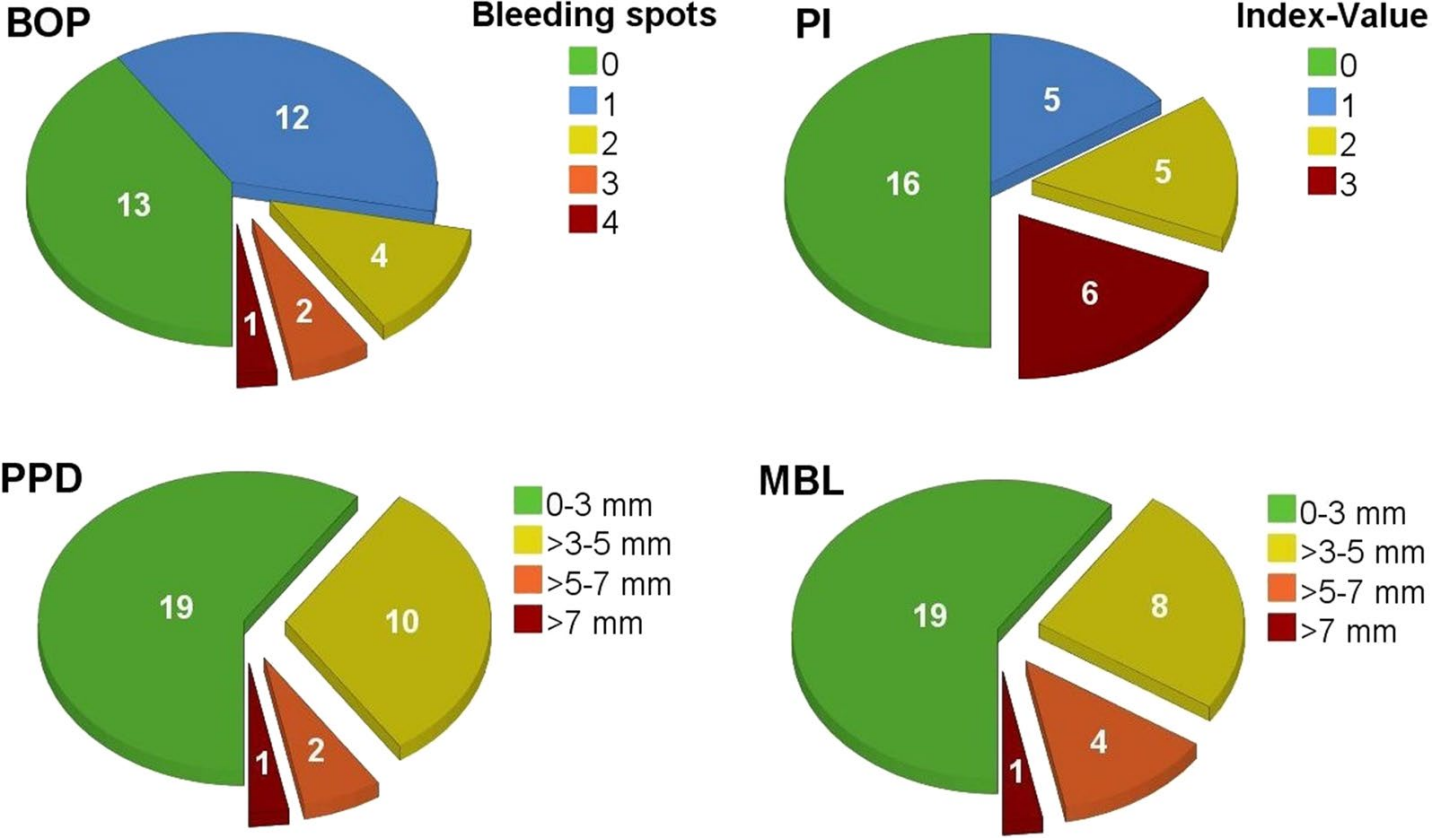
Frequency distribution on severity level of implant characteristics grouped into four circle diagrams for each parameter: Plaque Index (PI); Bleeding on Probing (BOP); Pocket Probing Depth (PPD); and Marginal Bone Loss (MBL) after a mean observation time of 29 years in CEG (32 implants). Diagrams for PI, PPD and MBL show the highest measured value for each implant

The overall average PPD was 2.4 ± 1.5 mm. Mean values for BOP and Plaque Index were 0.9 ± 1.1 and 1.0 ± 1.2, respectively. The frequency distribution of the severity levels of these parameters is shown in Fig. 5. A total of 3 implants were diagnosed with peri-implantitis leading to a peri-implantitis rate of 9.4%, which corresponds to an overall success rate of 90.6%.

### Marginal bone levels

Seven out of the 15 baseline radiographs were taken immediately after implant placement or within one week after insertion. For the current radiographic analysis, in one case, we had to resort to a radiograph not taken at follow-up. The mean loss of marginal bone was 2.5 ± 1.5 mm at the mesial implant site and 2.5 ± 2.1 mm at the distal implant site corresponding to a global average marginal bone loss of 2.5 ± 1.8 mm (Table 2). There was no significant difference between marginal bone loss at the mesial and distal implant sites (*P* = *0.6*). Importantly, the inter-rater variations were not significant for linear bone-level measurements (*P* = *0.9*). The inter-rater class coefficient was 1.0, indicating very good inter-rater conformity [14, 15]. The frequency distribution of the severity of marginal bone-level changes is shown in Fig. 5.

**Table 2:** Descriptive statistics

	*N* (implants)	Minimum	Maximum	Mean	Std.-deviation	Variance
Observation time (years)	32	23.50	34.80	28.89	3.24	10.49
Bleeding-on-probing Index (number of bleeding points)	32	0	4	0.94	1.05	1.09
Plaque Index (according to Mombelli, highest value)	32	0	3	1.03	1.20	1.45
Pocket probing depth mesial (mm)	32	1	7	2.91	1.65	2.73
Pocket probing depth distal (mm)	32	1	7	2.72	1.69	2.85
Pocket probing depth buccal (mm)	32	1	7	1.88	1.43	2.05
Pocket probing depth oral (mm)	32	1	11	2.19	1.86	3.45
Mean pocket probing depth (mm)	32	1.00	8.00	2.41	1.48	2.20
Width attached mucosa (mm)	28	0.00	5.00	1.11	1.69	2.84
Marginal bone loss mesial (mm)	32	0.30	6.95	2.47	1.75	3.06
Marginal bone loss distal (mm)	32	0.10	7.30	2.52	2.08	4.33
Mean marginal bone loss (mm)	32	0.38	7.13	2.51	1.84	3.38

### Correlation analysis

The statistical analysis identified significant positive correlations between “MBL” and “PPD” (*P* = *0.032*) as well as between both just mentioned parameters and “BOP “ (*P* = *0.006 and P* < *0.001,* respectively) indicating that more pronounced marginal bone loss is associated with higher pocket probing depths and more bleeding on probing. A significant but weak linear correlation was identified between “MBL” and “observation time” (*P* = *0.017*). According to the Shapiro–Wilk test, all values of the evaluated parameters showed a nonparametric distribution apart from “observation time”, as the significance levels for this parameter did not differ between the nonparametric Spearman-Rho and the parametric test according to Pearson. Table 3 shows the correlation coefficients according to Spearman-Rho.

**Table 3:** Correlation analysis according to Spearman-Rho in the CEG (32 implants)

	BOP	MBL	PPD
Observation time	0.022	0.017	
Plaque Index			
PPD	< 0.001	0.032	
MBL	0.006		
BOP			

The table exclusively highlights the *p*-values of the statistically significant findings

### Risk factors

In the CEG, two patients had been diagnosed with diabetes, five with osteoporosis, and seven with periodontitis. Four patients had a smoking habit, of which three also had periodontitis (Table 4). The proportions of smokers and diabetics in the CEG and CG were nearly identical, while the percentages of patients with osteoporosis and diabetes (33–21% and 47–38%, respectively) were notably higher. At the implant level, only one implant in each group of smokers, patients with diabetes and patients with periodontitis was associated with peri-implantitis, while patients with osteoporosis did not exhibit any compromised implant. No significant correlation between the described risk factors and the presence of peri-implant disease was identified in the Chi-squared test.

**Table 4:** Cross-table showing the association between patient risk factors and the presence of peri-implant disease

	Implants diagnosed with peri-implantitis (number of corresponding patients)		
	Yes	No	Total
*Smoking habit (P = 0.726)*			
Yes	1 (1)	7 (4)	8
No	2 (2)	22 (11)	24
Total	3	29	32
*Diabetes (P = 0.252)*			
Yes	1 (1)	3 (2)	4
No	2 (2)	26 (13)	28
Total	3	29	32
*Osteoporosis (P = 0.420)*			
Yes	0 (0)	10 (5)	10
No	3 (3)	18 (10)	21
Total	3	28	31*
*Periodontitis (P = 0.900)*			
Yes	1 (1)	12 (7)	13
No	2 (2)	16 (8)	18
Total	3	28	31*
*Type of restoration (P = 0.702)*			
Fixed	1 (1)	13 (7)	14
Removable	2 (2)	16 (8)	18
Total	3	29	32

Due to the limited number of implants with peri-implantitis, no significant correlation could be shown

^*^Medical history regarding osteoporosis and periodontitis was missing for one patient

### Fixed vs. removable prostheses

**Fig. 6:**
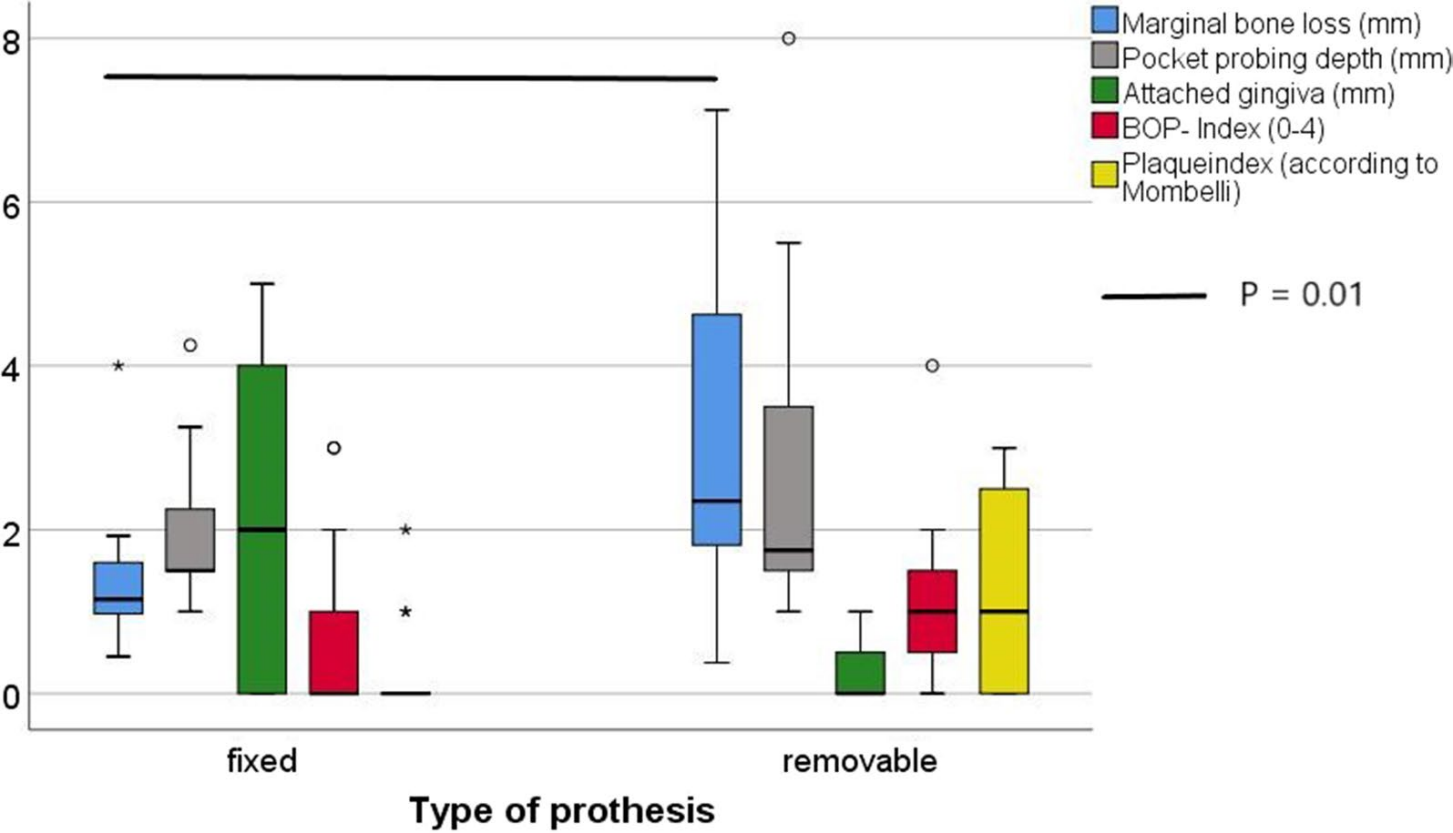
Comparison of clinical and radiographic findings categorized in two groups according to type of dental prosthesis. Statistically significant differences were identified solely for the parameter “marginal bone loss”

Out of the 32 implants, 14 implants in seven patients were treated with fixed dental prostheses (FDPs), while 18 implants in eight subjects were treated with removable dental prostheses (RPDs). Differences in clinical and radiographic findings between implants in the FDPs and RDPs groups were found for MBL with the *t*-test and Mann–Whitney *U* test (*P* = 0.02 and *P* = 0.01, respectively). While the mean marginal bone-level change for implants in the FDPs group was 1.7 ± 1.3 mm, MBL for implants in the RDPs group amounted to 3.2 ± 2.0 mm. Further comparisons of the clinical parameters showed no significant differences in PPD and BOP between the two groups (Fig. 6). Despite being statistically significant, differences in the PI and width of the attached mucosa could not be compared properly because of major differences in the variance of the parameter values. Regarding peri-implantitis, one implant in the FDPs group and two implants in RDPs group were compromised. The analysis with the Chi-square test showed no significant correlation (*P* = 0.702).

## Discussion

Of the 94 preselected and possible participants in this study, 30.9% passed away, and 27.6% were unavailable, leading to a positive response rate of 41.5% (CG). A further 25.5% of these patients had to be excluded for the various reasons listed in Table 5, ultimately leaving 16% of the patients from our original cohort who were examined after a mean observation time of 28.3 years. Comparable long-term studies with observation periods of up to 25 years showed dropout rates ranging from 31.6–71.7%, which is significantly lower than the 84% dropout rate in our study [16–26]. Variations in observation periods may be a decisive factor for this discrepancy, as our study had a longer mean observation time. Thus, on average, patients in our study cohort were older, leading to a higher number of deaths (Table 5). Only one previous study by Bakker et al. (2019) included a cohort whose average age (85.5 + years) significantly surpassed the mean age found in our study, as patients younger than 60 years at baseline were excluded [17]. Consequently, the proportion of patient deaths (62.3%) in this cohort was the highest, leading to a dropout rate of 71.7%. The relatively small number of “unavailable” patients compared to our study might be explained by its prospective study design, since those patient cohorts have been meticulously followed up several times over the course of the last 20 years [17]. Generally, dropout rates are very high, with most studies observing at best, about 30%, and more often about 60% after 20 years, regardless of the study design. Importantly, cutting out such a large proportion of potential information limits the validity of the collected data.

**Table 5:** Follow-up studies providing data on implant therapy after more than 15 years

Authors	Design	Mean observation time (years)	Patient selection	Implant system	Type of restoration	Number of patients/implants	Drop-out (% patients/% implants)	Reasons for Drop-out	Patient’s mean age at the time of examination (years)	Survival rate (implants)
Dierens et al. (2011)	Retrospective	18.4	- Treated between 1987 and 1993 - Availability of peri-apical radiographs - Availability of original patient file	Branemark	Single crown (SC)	134/166	84/104 (62.7%/62.7%)	- Deceased (4) - No contact information (9) - Moved away (11) - Did not answer phone (2) - Loss of all original implants (7) - Unwilling to attend clinical examination (51)	42.4	91.5% (cumulative, contact group)
Frisch et al. (2020)	Retrospective	25.4	- Treated between 1991 and 1996 - Availability of radiographs after ≥ 20 years	Ankylos, Branemark, IMZ, ITI Bonefit	SC, FDP, RDP	62/?	36/? (58.1%/?)	- Deceased or serious disease (24) - No participation in supportive implant therapy program (SIT) (7) - No radiographs (1) - Reason not known (4)	74.6	94.7% (percentage implants lost)
Astrand et al. (2008)	Prospective	20	- Original study cohort - Edentulous patients in one or two jaws	Branemark	RDP	48/?	27/146 (56.3%/?)	- Deceased (19) - Age/sickness (8)	61–94 (range)	99.2% (percentage implants lost)
Lekholm et al. (2006)	Retrospective	20	- Treated between 1983 and 1985 - Treated and followed by one surgeon - Availability of baseline radiographs	Branemark	SC, FDP	27/112	10/43 (37%/38.4%)	- Deceased (6) - Moved away (3) - Unwilling to attend follow-up (1)	68	91% (cumulative, including drop out)
Jokstad et al. (2017)	Retrospective	17.5	- Implant treatment before 2002	Branemark	SC, FDP	298/?	177/? (59.4%/?)	- Could not be located (92) - Deceased (23) - Moved away (21) - Age/illness (14) - Unwilling to attend follow-up (23) - Loss of original implant(s) (4)	62	94% (percentage implants lost)
Chappuis et al. (2013)	Prospective	20	- Original study cohort - Partially edentulous patients	Bonefit	FDP	98/145	31/50 (31.6%/34.5%)	- Deceased (11) - Moved away, age, illness (20)	66.3	89.5% (percentage implants lost)
Bakker et al. (2019)	Prospective	20	- Original study cohorts (Batenburg et al., 1998; Heydenrijk et al., 2002) - Edentulous patients - Patient’s age > 60 years at the time of implant placement	Branemark, IMZ, ITI	RDP	53/106	38/76 (71.7%)	- Deceased (33) - Moved away (5)	85.5	92.5% (percentage implants lost, including drop out)
Deporter et al. (2014)	Prospective	20	-Original study cohort (Deporter et al. 1992) -Edentulous patients with advanced mandibular resorption	SPS	RDP	52/156	30/103 (57.7%/66%)	-All implants lost within the first 2 years (2) - Deceased (11) -Age, Illness (17)	Not specified	73.4% (cumulative, including drop out except the 2 patients with early loss of all implants)
Jacobs et al. (2010)	Prospective	16	-Original study cohort (van Steenberghe et al. 2000) -Patients with bilateral tooth loss (Kennedy Class 1) -Treated between 1993 and 1994	Astra, Branemark	FDP	18/95	6/48 (33.3%/50.5%)	-Deceased (1) -Moved away (3) -Age (2)	Not specified	97.7%-100% (cumulative success rate, including drop out, range between subgroups)
Jung et al. (2021)	Prospective, comparative	22–24 (mean value not described)	-Original study cohort (Zitzmann et al. 2001) -Treated from 1994–1996	Branemark, Biomed 3i, IMZ	Fixed, removable	72/265	33/118 (45.8%/44.5%)	-Deceased (23) -Moved away, illness (10)	75.8	89.3–93.8% (cumulative, range between subgroups)
Donati et al. (2018)	Prospective, comparative	20	-Original study cohort (Wennström et al. 2004)	Astra	FDP	51/148	26/84 (51%/56.8%)	-Deceased (19) -Moved away, illness (7)	Not specified	87.8% (percentage implants lost)
Our findings	Retrospective	28.3	- Treated between 1981–1995 - Availability of baseline radiographs	IMZ	FDP, RDP	94/199	79/167 (84%/83.9%)	- Deceased (29) - Unavailable (26) - Loss of all original implants (13) - Age/illness (5) - Moved away (4) - No up-to-date radiographs (2)	75.3	79.5% (contact group)

Owing to steadily evolving implant technology, the lack of sufficient long-term data is an issue, especially for outdated implant systems, as the benefit of data on those systems is arguable. Furthermore, the presented studies indicate that the feasibility of long-term follow-up studies spanning 20 years or longer is complicated due to high patient dropout rates [16–18, 20, 22, 24, 26].

The present study showed a relatively high survival rate of 79.5% after a mean observation time of 28 years. However, it falls short of the survival rates of follow-up studies with comparable observation periods, ranging from a survival rate between 87.8. and 100% [16–18, 20–26]. Interestingly, studies exclusively examining Branemark implants have reported the highest survival rates, regardless of the study design. On the other hand, Frisch et al. (2020) observed excellent survival rates in a cohort of patients with various implant types, who were part of a permanent supportive implant therapy program [22]. Nevertheless, due to the heterogeneity of study designs and the generally high dropout rates described in all studies to date, the results should be interpreted with caution.

Regarding the radiographic analysis, we found a mean marginal bone loss of 2.5 mm after an average observation time of 28.3 years. While slightly higher, our results are comparable to those of previous studies [16, 17, 20, 22–26] reporting mean marginal bone loss in the range of 0.02–2.5 mm analyzing predominantly Branemark implants (Table 6). Regarding clinical outcomes, we recorded an average probing pocket depth of 2.4 mm. In relation to the mean pocket probing depths ranging from 2.5 to 4.0 mm in comparable studies (Table 6), our results stand out in a positive way. Owing to the variability of PPD which depends on the width of the peri-implant mucosa, information on the progression or stagnation of PPD is essential for peri-implant diagnostics. In addition, our favorable findings can be explained by the reduced accessibility of the peri-implant pocket due to the geometry of the implant’s suprastructure [27], as in our study the implant suprastructures were not removed for clinical evaluation.

**Table 6:** Mean values for MBL, PPD, plaque, and Bleeding Index as well as the corresponding peri-implantitis rate, as described by the authors in Table 5

Authors	Mean marginal bone loss (mm)	Mean pocket probing depth (mm)	Mean value Plaque Index	Mean value Bleeding Index	Peri-implantitis rate
Dierens et al. (2011)	1.7 ± 0.88 (from baseline)	3.9 ± 1.27	0.25 ± 0.35 (acc. to Loe, 1967)	Not specified	Not specified
Frisch et al. (2020)	1.8 ± 1.2 (bone level)	3.69 ± 1.06	Not specified	36.6% (implants with BOP)	7%, 40% (incidence)
Astrand et al. (2008)	1.72 ± 0.16 (from baseline)	3.4	Not specified	Not specified	2.4%
Lekholm et al. (2006)	1.0 (from baseline)	Not specified	Not specified	Not specified	Not specified
Jokstad et al. (2017)	1.52 ± 1.57 (bone level)	2.5 ± 1.3	0.8 ± 0.98 (acc. to Mombelli, 1987)	0.92 ± 0.83 (acc. to Mombelli, 1987)	5.1%
Chappuis et al. (2013)	3.04 (bone level, median)	3.14 ± 0.95	0.44 ± 0.64 (acc. to Mombelli, 1987)	0.11 ± 0.41 (acc. to Mombelli, 1987)	13.7% (incidence)
Bakker et al. (2019)	1.14 ± 0.85 (from baseline)	3.5 (median)	2 (median, acc. To Mombelli, 1987)	1 (median, acc. to Mombelli, 1987)	Not specified
Deporter et al. (2014)	0.67 (bone level)	Not specified	Not specified	Not specified	Not specified
Jacobs et al. (2010)	0.02 ± 0.45–0.31 ± 0.69 (from baseline)	Not specified	Not specified	Not specified	Not specified
Jung et al. (2021)	2.0 ± 1.4–2.5 ± 1.5 (from baseline)	2.9 ± 1.1–3.3 ± 1.1	6–21% (acc. to O’Leary)	34–41% (BOP)	Not specified
Donati et al. (2018)	0.41 ± 1.25–0.83 ± 1.59 (from baseline)	3.7 ± 1.03–4.0 ± 1.3	14.8–25.9% (index not specified)	11.1–25.9% (BOP)	10.9%
Our findings	2.5 ± 1.8 (from baseline)	2.4 ± 1.5	1.0 ± 1.2 (acc. to Mombelli, 1987)	40.6% (implants with BOP)	9.4%

Comparing the MBL of the IMZ implants among the FDPs and RDPs groups, we found significantly less bone-level changes in the FDPs group (1.7 mm and 3.2 mm, respectively). To date, no studies have analyzed bone-level changes in IMZ implants in fixed dental prostheses. Regarding the MBL of IMZ implants in removable prostheses, we observed significantly higher bone loss than the 1.4 mm described by Meijer et al. (2009) after 10 years. Therefore, owing to the vast differences in observation times, comparisons must be performed with caution. Notably, based on differences observed in MBL between the FDPs and RDPs groups, Berglundh et al. (2002) concluded, that the percentage of implants showing MBL of at least 2.5 mm is notably higher (4.76%) in overdentures than in FDPs or single-implant restorations (1.01% and 1.28%, respectively) [28]. These results confirm our findings. The increased bone loss might be explained by the unfavorable bending forces that are applied to the implants due to the free-ending saddles typically used in RDPs [29].

Of the 32 implants included in our study, 3 (9.4%) were diagnosed as peri-implantitis, which is a slightly higher prevalence rate than that reported in comparable studies ranging from 2.4–7% [16, 22, 24]. However, the value is in accordance with the 10% proportion of peri-implant diseased implants reported in the review by Mombelli et al. (2012) after an observation time of 5–10 years [30]. As pointed out in the review by Mombelli et al. (2012), a wide range of different disease-defining criteria were used in the selected studies, so the comparability of peri-implantitis rates is generally difficult. For example, according to Astrand et al. (2008), a crater or beaker-like type of bone loss is required to diagnose peri-implantitis [16], while Jokstad et al. (2017) defined diseased implants as unsuccessful implants according to the criteria of Buser et al. (1990) [24, 31].

Due to these different definitions, the 2017 Consensus Conference established a uniform standard. Consequently, the new definition for peri-implantitis was used in the current study. As one of the latest long-term follow-ups on implant therapy Frisch et al. (2020) also followed this new standard allowing a more concise comparison of the presented per-implantitis rates [22]. Frisch et al. (2020) observed a prevalence of peri-implantitis of 7% in a cohort of patients participating in a strict supportive implant therapy program. In contrast, a peri-implantitis incidence rate of 40% was observed among patients that were not part of a supportive structured implant therapy program but instead kept control examination appointments on their own initiative. In contrast to these data, the results of our study seem very favorable, even if on our patient cohort a strict supportive implant therapy program was not performed. Further, because of the absence of previous examinations in contrast to Frisch et al. (2020) we had to rely on the thresholds proposed in the Consensus report [13] limiting the comparability between the presented peri-implantitis rates. Due to the geometry of the implant’s suprastructure, the access to the peri-implant pocket was reduced possibly leading to a more positive outcome, as suprastructures were not removed in our study [27]. Nevertheless, a recent systematic review also observed significant differences in the prevalence of peri-implantitis between patients who participated regularly in a prophylaxis program (9.0%) and those without regular preventive maintenance care (18.8%). These findings confirm a tendency towards an increased risk for developing peri-implantitis in patients with a lack of prophylaxis on a medium level of evidence [32]. Although several studies have found a strong tendency to favor peri-implantitis in smokers, patients with periodontitis and patients with diabetes [30, 32], our study failed to show a significant connection between these risk factors and the development of peri-implantitis. As we predominantly relied on self-reported medical history, it is most likely that many patients did not know or remember the exact reason for their teeth loss leading to implant therapy. In our study, 46.7% of the patients had previously suffered from periodontitis, although the actual number might be greater, considering that the fifth German oral health study reported that 65% of elderly people suffer from periodontal disease, which is considerably higher than that observed in our study [33]. With regard to osteoporosis as a potential risk factor for peri-implantitis, neither our study nor the systematic review by Dreyer et al. (2018) found any significant associations [32].

Our literature search in MEDLINE did not reveal any meta-analysis nor systematic review on implant survival and marginal bone loss after an observation time of 15 years or longer. This applies not only to IMZ implants but also in general to other implant systems. Latest systematic reviews on this topic present data on implant therapy after 10 years, while studies with longer follow-up periods were occasionally included [34, 35]. In these studies, implant survival ranges from 73.4 to 100% with a cumulative mean value of 94.6% [35].

According to the aims of the presented study, we compiled publications analyzing patient drop out, implant survival and peri-implant conditions with long-term data after more than 15 years (Tables 5, 6). We included all studies with a prospective as well as retrospective design. Publications not providing a distinct description of patient drop out were excluded. The selected studies partially providing detailed investigation on long-term implant therapy suggest that a systematic review and meta-analysis on this topic in a dedicated publication is meaningful.

The major limiting factor of our study was the small number of patients, who attended the follow-up appointment. Of the 94 patients with 199 implants, 15 patients with 32 implants agreed to undergo clinical and radiographic examinations. This was equivalent to a dropout rate of 84%. Since most of the dropped-out patients were deceased or unavailable, and the patient’s status was considered independent of the condition of the implants, we assume that our contact group is representative of the original patient pool. However, it is arguable whether patients with CEG were generally healthier and therefore more compliant than patients who were not available for a follow-up appointment because of senility or death. Additionally, as the clinical and radiographical data of lost implants could not be considered, our results are probably based on a positive selection of largely successful implants. Despite these assumptions, the influence of a largely reduced study population accompanied by a corresponding loss of information is unclear and could equally lead to false-positive or false-negative outcomes, which is why the results of our study should be interpreted with caution. This applies especially to the results from the radiographic analysis. Although differences in the reported bone loss across all the studies mentioned are minimal, strict comparisons are difficult, and exact conclusions cannot be drawn. As seen in our study, the number of patients examined at follow-up after 10–20 years was comparably small. Hence, individual findings and statistical outliers could drastically affect the results, leading to greater variability in the reported outcomes.

In addition, one major problem in analyzing the radiographs was the rather difficult identification of the reference points of the implant according to our definition. Since it was not possible to remove the implant suprastructures for examination, the radiographic reference points were often overlaid by the intramobile connector (IMC). In addition, radiographic superimposition and motion blur led to varying image quality and in some cases, the position of the radiographic reference point had to be gauged. The bone-level measurements were performed by two individual examiners with the help of optical magnification, thus any possible deviations between the estimated and actual reference points should be minor and clinically irrelevant.

Due to the retrospective nature of this study, combined with missing or incomplete patient files, no statements on changes in clinical parameters could be made, as no baseline data were reported. In addition, we often had to rely on self-reported medical history, limiting the validity of the presented analysis of risk factors, as the senescence of the patients has to be considered. The lack of memory and understanding for their anamnesis could explain the relatively small number of patients with a history of periodontitis found in our study.

## Conclusions

Conducting long-term follow-up studies with a retrospective design after a mean observation time of nearly 30 years with acceptable response rates was not feasible. Therefore, owing to the high dropout rate of 84%, the results cannot be generalized and should be interpreted with caution. The limited data we could gather presented an overall implant survival rate of 79.5%, while the surviving implants showed a peri-implantitis rate of 9.4%, exhibiting a mean marginal bone loss of 2.5 mm. Due to the retrospective study design and the high dropout rate, additional risk factors could not be considered in a conclusive analysis.

## Data Availability

The datasets used and/or analyzed during the current study are available from the corresponding author on reasonable request.

## Abbreviations

IMZ: Intramobile cylinder
IME: Intramobile element
IMC: Intramobile connector
CG: Contact group
CEG: Clinical evaluation group
PPD: Pocket probing depth
BOP: Bleeding on probing
PI: Plaque Index
MBL: Marginal bone loss
FDP: Fixed dental protheses
RDP: Removable dental protheses
